# Docosahexaenoic acid improves behavior and attenuates blood–brain barrier injury induced by focal cerebral ischemia in rats

**DOI:** 10.1186/s13231-014-0012-0

**Published:** 2015-01-28

**Authors:** Sung-Ha Hong, Larissa Khoutorova, Nicolas G Bazan, Ludmila Belayev

**Affiliations:** Neuroscience Center of Excellence, Louisiana State University Health Sciences Center, 2020 Gravier Street, Suite D, New Orleans, LA 70112 USA; Department of Neurosurgery, Louisiana State University Health Sciences Center, 2020 Gravier Street, Suite D, New Orleans, LA 70112 USA

**Keywords:** Experimental stroke, Behavior, Blood–brain barrier, Evans Blue, FITC-dextran, Neuroprotection

## Abstract

**Background:**

Ischemic brain injury disrupts the blood–brain barrier (BBB) and then triggers a cascade of events, leading to edema formation, secondary brain injury and poor neurological outcomes. Recently, we have shown that docosahexaenoic acid (DHA) improves functional and histological outcomes following experimental stroke. However, little is known about the effect of DHA on BBB dysfunction after cerebral ischemia-reperfusion injury. The present study was designed to determine whether DHA protects against BBB disruption after focal cerebral ischemia in rats.

**Methods:**

Physiologically-controlled SD rats received 2 h middle cerebral artery occlusion (MCAo). DHA (5 mg/kg) or vehicle (saline) was administered I.V. at 3 h after onset of MCAo. Fluorometric quantitation of Evans Blue dye (EB) was performed in eight brain regions at 6 h, 24 h or 72 h after MCAo. Fluorescein isothiocynate (FITC) - dextran leakage and histopathology was evaluated on day 3 after stroke.

**Results:**

Physiological variables were stable and showed no significant differences between groups. DHA improved neurological deficits at 24 h, 48 h and 72 h and decreased EB extravasation in the ischemic hemisphere at 6 h (by 30%), 24 h (by 48%) and 72 h (by 38%). In addition, EB extravasation was decreased by DHA in the cortex and total hemisphere as well. FITC-dextran leakage was reduced by DHA treatment on day 3 by 68% compared to the saline group. DHA treatment attenuated cortical (by 50%) and total infarct volume (by 38%) compared to vehicle-treated rats on day 3 after stroke.

**Conclusions:**

DHA therapy diminishes BBB damage accompanied with the acceleration of behavioral recovery and attenuation of the infarct volume. It is reasonable to propose that DHA has the potential for treating focal ischemic stroke in the clinical setting.

## Introduction

Cerebral ischemia can cause blood–brain barrier (BBB) disruption and increased cerebral vascular permeability, leading to the formation of brain edema [1,2]. Clinically, BBB disruption occurs in more than one third of stroke patients and is associated with poor outcomes and lower survival rates following stroke [3,4]. In animal models of cerebral ischemia–reperfusion, BBB disruption is believed to be biphasic, which implies an early first opening (at 3–6 h) followed by a refractory period when the BBB is closed, and a delayed second opening (at 48–72 h) [5,6]. Since cerebral ischemia and BBB damage are closely correlated, so approaches for protecting BBB integrity and reducing BBB permeability could help elucidate the pathophysiological mechanism in brain ischemia, leading to novel therapies as well as providing a mechanistic roadmap to evaluate efficacy of treatment [7].

Recently, we have shown that docosahexaenoic acid (DHA; 22:6, n-3), a member of the essential omega-3 fatty acid family, improves functional and histological outcomes in experimental stroke [8,9]. DHA is highly concentrated in the brain and is involved in cognition and other brain functions [10,11]. DHA is the precursor of the docosanoids, and neuroprotectin D1 (NPD1; 10R,17S-dihydroxy-docosa-4Z,7Z,11E,15E,19Z hexaenoic acid), being the first identified member of this group of mediators, inhibits oxidative stress-induced proinflammatory gene expression and promotes cell survival [12,13]. DHA is involved in excitable membrane function, neuronal signaling and has been implicated in neuroprotection [8,9]. DHA administration improves histological and neurological outcome following focal cerebral ischemia in rats when treatment is initiated as late as 5 hours after ischemia onset [9].

Many methods for assessing the disruption of the blood–brain barrier have been adopted in experimental models [14]. Small (EB, 68 kDa) and large (FITC-dextran, 2000 kDa) tracers were used here to evaluate BBB permeability. Although distribution of these two markers are similar (primarily, if not exclusively, extracellular), intracellular and extracellular uptake of EB was reported [15]. Serum albumin-bound EB crosses the easily broken BBB, and its regional fluorometric quantification provides the precise assessment of the degree of BBB disruption in ischemic injury [16]. Subsequently, the presence or absence of highly localized extracellular FITC-dextran leakage has been used to identify brain regions undergoing vascular disruption and to label the severely damaged BBB [15,17]. The present study was designed to determine whether DHA protects against BBB disruption after focal cerebral ischemia in rats.

## Materials and methods

### Animal preparation

The present study was conducted in accordance with the NIH guidelines for the care and use of animals in research and under protocols approved by the Institutional Animal Care and Use Committee of the Louisiana State University Health Sciences Center, New Orleans. Male Sprague–Dawley (SD) rats (3**–**4 months-old); Charles River Laboratory, Wilmington, MA) were fasted overnight with free access to water prior to the surgical procedure. Anesthesia was induced by the inhalation of 3% isoflurane in 70% NO and 30% O_2_ mixed gases, and then maintained with 1% isoflurane in the same mixed gases during the procedure. Orally-intubated animals were mechanically ventilated after the immobilization by injection of pancronium bromide (0.5 mg/kg, I.V.). The catheters were implanted into the right femoral artery and vein for the blood sampling and infusion of drug, EB and FITC-dextran. Serial analyses of arterial blood gases and plasma glucose were conducted before and during surgical procedure. Rectal (CMA/150 Temperature Controller, CMA/Microdialysis AB, Stockholm, Sweden) and cranial (temporalis muscle; Omega Engineering, Stamford, CT) temperatures were closely monitored before, during and after MCAo. Rectal temperature and body weight were monitored daily and before sacrifice during the survival period.

### Transient Middle Cerebral Artery occlusion (MCAo)

The right MCA was occluded for 2 h by intraluminal filament, as we described previously [18]. Briefly, the right common carotid artery (CCA) and external carotid artery (ECA) were exposed through midline neck incision, and then completely isolated from the surrounding nerves. The occipital branches of the ECA and pterygopalatine artery were ligated. A 4-cm of 3–0 nylon filament, coated with poly-L-lysine was advanced to the origin of MCA through the proximal ECA via internal carotid artery. The filament was inserted 20 to 22 mm from the bifurcation of the CCA, according to the animal’s body weight. The neck incision was then closed and rats were returned to their cages. After 2 h of MCAo, the rats were re-anesthetized with the same anesthetic combination and the intraluminal filament was gently removed. The animals were allowed to survive for different times according the experimental protocol with free access to water and food.

### Behavioral tests

Behavioral tests were conducted before, during MCAo (at 60 min), and then at 6, 24, 48 and 72 h after MCAo by an investigator who was blinded to the experimental groups. The battery consisted of two tests, (1) postural reflex to examine the upper body posture when the rat was suspended by tail, and (2) forelimb placing test to assess the forelimb placing responses to visual, tactile and proprioceptive stimuli [18]. Neurologic function was graded on a scale of 0 to 12 (normal =0, maximal deficits = 12), as we described previously [18]. The severity of stroke injury was assessed by behavioral examination of each rat at 60 min after onset of MCAo. Rats that did not demonstrate high-grade contralateral deficit (score, 10–11) were excluded from further study. Two animals were excluded for this reason.

### Treatment groups

Animals were randomly assigned to DHA (5 mg/kg, Cayman, Ann Arbor, MI) or vehicle (0.9% saline) treatment groups. All treatments were administered intravenously at 3 h after the onset of MCAo at a constant rate over 3 min using an infusion pump. The DHA dose–response study in rats with transient focal cerebral ischemia showed that a 5 mg/kg dosage was highly neuroprotective [8]; thus this dose was applied in this study.

### Study protocols

Two protocols were used. In series 1, BBB leakage was studied by EB dye. The neurological status was evaluated at 6, 24 and 72 h followed by EB measurement at 6, 24 or 72 h (n = 8-12 rats per group). In series 2, BBB leakage was assessed by FITC-dextran. Behavioral score was measured on days 1, 2 and 3. On Day 3, FITC-dextran leakage was analyzed followed by histopathology (n = 5-7 rats per groups). EB, FITC and histopathological analyses were conducted by an investigator who was blinded to the experimental groups.

### Evaluation of BBB leakage by Evans Blue (EB) dye

**Figure 1:**
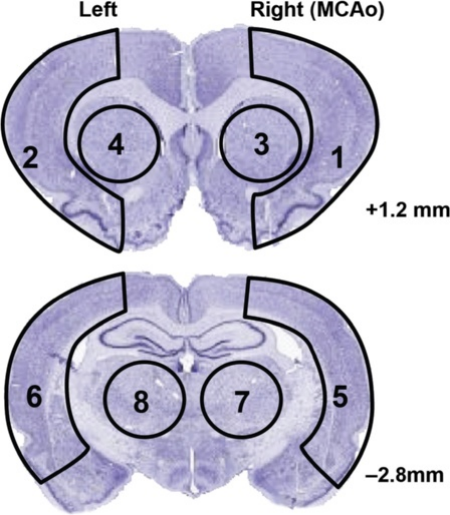
**Diagram of brain sampling for EB study.** Coronal sections of rat brain illustrating division of right **(R)** and left **(L)** hemispheres into eight regions for measurement of tissue Evans Blue dye. Coronal levels with references to bregma are noted.

The integrity of the BBB was investigated using EB extravasation [19]. EB (2%, 4 ml/kg, in saline, Sigma-Aldrich, MO) was injected intravenously either at 5 h, 23 h or 71 h after MCAo. After 1 h of EB circulation, rats were anesthetized with isoflurane and were transcardially perfused with saline until colorless fluid was observed from the right atrium. After decapitation, the removed brains were divided into two coronal blocks that included the bregma levels + 0.3 and – 1.8 mm. Coronal blocks were divided into right and left hemisphere, and were cut into eight regions for local measurement of EB dye (see Figure 1). EB extravasation into the brain was measured as we previously described [19]. Samples were weighed and placed in 50% of tricholoroacetic acid. Following homogenization and centrifugation at 10000 RPM for 20 min, extracted EB dye was diluted with ethanol (1:3) and its fluorescence was determined (excitation at 620 nm and emission at 680 nm) by a Spectra Max M5e (Molecular Devices, CA). External standards were prepared in a range of 100 – 500 ng/ml in the same solvent. The tissue content of EB was calculated based on the linear standard curve and expressed as μg of EB/g of tissue.

### Evaluation of BBB leakage by fluorescein isothiocyanate dextran (FITC-dextran)

FITC-dextran (2000 kDa, 1 ml, 50 mg/ml, in saline, Sigma-Aldrich, St. Louis, MO) was intravenously injected at 72 h after MCAo [20,21]. After 1 min of circulation, brains were removed, fixed in 4% paraformaldehyde for 24 h, cryoprotected in 30% sucrose for 48 h and then coronally sectioned (20 μm-thickness) using the cryostat. Mosaic fluorescent images of the sections were obtained at six bregma levels (+2.7, + 1.2, − 0.3, − 1.3, − 1.8 and - 3.8 mm) with fluorescence motorized microscope (BX61VS, Olympus, Japan) at a 10 X magnification objective lens. Regional FITC-dextran fluorescent intensity in the ipsi- and contra-lateral cortex, subcortex and hemisphere were measured by Image J (National Institute of Health, Bethesda, MD). Optical density was measured by applying the following formula: (Right fluorescent intensity – Left fluorescent intensity)/Left fluorescent intensity × 100.

### Histopathology

Histopathology was performed after the evaluation of BBB leakage by FITC-dextran at 72 h as we previously described [18]. Adjacent coronal sections (20 μm-thickness) from FITC-dextran study were stained with thionine (Nissl). Stained sections at nine standardized bregma levels were digitized, and then cortical, subcortical infarct areas were outlined and measured as well as the both hemispheres using MCID core imaging software (InterFocus Imaging Ltd., Cambridge, England). Infarct volume was quantified as an integrated product of cross-sectional infarct areas and intersectional distance and corrected for brain swelling. Computer generated mosaic images of stained sections were obtained by motorized microscope (BX61VS, Olympus, Japan) at a 10 X magnification objective lens.

### Statistical analysis

Repeated-measures analysis of variance (ANOVA), followed by Bonferroni tests, was used for multiple-group comparisons. Two-tailed Student’s *T*-test was used for two-group comparisons. A value of p < 0.05 was regarded as statistically significant. Values are presented as means ± SEM. Statistical analyses were conducted by Prism 5 (Graph Pad Software, Inc., La Jolla, CA).

## Results

There were no significant differences in the rectal and cranial (temporalis muscle) temperatures, body weight, arterial blood gases, plasma glucose and hematocrit among the groups (Table 1). Four animals died during the experiment: 3 rats in the saline group (on days 1, 2 and 3) and 1 rat in the DHA group (on day 1).

**Table 1:** **Physiological variables**

	**Evans-Blue Study**						**FITC-dexran Study**	
	**Saline - 6 h**	**DHA - 6 h**	**Saline - 24 h**	**DHA - 24 h**	**Saline - 72 h**	**DHA - 72 h**	**Saline - 72 h**	**DHA - 72 h**
	**(n=9)**	**(n=9)**	**(n=9)**	**(n=8)**	**(n=12)**	**(n=12)**	**(n=7)**	**(n=6)**
**Before MCAo (15min)**								
Cranial Temperature (°C)	37.2 ± 0.1	37.4 ± 0.1	37.1 ± 0.1	37.1 ± 0.2	37.4 ± 0.1	37.2 ± 0.1	37.2 ± 0.2	37.2 ± 0.2
Rectal Temperature (°C)	37.2 ± 0.1	37.3 ± 0.1	37.2 ± 0.1	37.3 ± 0.2	37.3 ± 0.1	37.2 ± 0.1	37.0 ± 0.1	37.0 ± 0.2
pH	7.49 ± 0.0	7.53 ± 0.0	7.50 ± 0.1	7.47 ± 0.0	7.47 ± 0.0	7.53 ± 0.0	7.4 ± 0.1	7.5 ± 0.0
pO2 (mmHg)	113.3 ± 4.1	120.7 ± 4.6	113.8 ± 6.8	114.9 ± 5.0	119.8 ± 3.7	120.4 ± 4.2	126.3 ± 8.5	113.7 ± 0.7
pCO2 (mmHg)	39.3 ± 0.4	38.7 ± 0.7	40.1 ± 0.6	38.4 ± 0.8	39.0 ± 0.5	38.1 ± 0.6	39.1 ± 0.9	38.3 ± 5.1
Plasma Glucose (mg/dL)	167.1 ± 12.7	171.2 ± 8.8	166.3 ± 9.9	194.9 ± 12.7	171.1 ± 13.0	161.7 ± 8.0	196.9 ± 6.1	204.7 ± 13.2
Hematocrit (%)	45.7 ± 12.7	45.8 ± 0.9	44.4 ± 0.8	45.8 ± 0.5	57.2 ± 11.7	45.8 ± 0.6	45.6 ± 0.6	45.8 ± 1.0
**During MCAo (15 min)**								
Cranial Temprature (°C)	37.6 ± 0.1	37.6 ± 0.1	37.4 ± 0.1	37.3 ± 0.1	37.7 ± 0.1	37.2 ± 0.1	37.6 ± 0.2	37.6 ± 0.1
Rectal Temperature (°C)	37.6 ± 0.1	37.4 ± 0.1	37.5 ± 0.1	37.5 ± 0.1	37.7 ± 0.1	37.5 ± 0.1	37.4 ± 0.1	37.3 ± 0.1
pH	7.51 ± 0.1	7.51 ± 0.0	7.46 ± 0.0	7.48 ± 0.0	7.46 ± 0.0	7.52 ± 0.0	7.5 ± 0.1	7.6 ± 0.1
pO2 (mmHg)	109.7 ± 4.4	114.0 ± 4.3	106.0 ± 4.8	106.9 ± 2.7	109.3 ± 3.4	114.6 ± 4.0	108.1 ± 4.9	105.0 ± 4.3
pCO2 (mmHg)	39.8 ± 0.3	39.8 ± 0.8	39.6 ± 0.6	39.0 ± 0.4	38.8 ± 0.4	38.5 ± 0.5	39.6 ± 0.7	39.2 ± 0.8
Plasma Glucose (mg/dL)	167.0 ± 10.6	164.0 ± 4.4	164.0 ± 4.6	193.3 ± 13.0	173.2 ± 15.8	155.0 ± 5.1	185.7 ± 6.9	197.3 ± 12.5
Hematocrit (%)	45.2 ± 0.6	46.3 ± 0.1	44.3 ± 0.8	45.6 ± 0.3	57.2 ± 11.7	46.0 ± 0.6	44.7 ± 0.8	46.7 ± 1.0
**After treatment (15 min)**								
Cranial Temprature (°C)	37.4 ± 0.1	37.1 ± 0.1	37.2 ± 0.2	37.1 ± 0.2	37.2 ± 0.2	37.0 ± 0.2	36.7 ± 0.2	36.9 ± 0.2
Rectal Temperature (°C)	37.7 ± 0.1	37.4 ± 0.2	37.8 ± 0.2	37.9 ± 0.2	37.7 ± 0.2	37.5 ± 0.1	36.9 ± 0.2	37.4 ± 0.2
**After treatment (24 h)**								
Rectal Temperature (°C)			38.2 ± 0.3	37.9 ± 0.3	37.4 ± 0.9	38.0 ± 0.2	37.8 ± 0.3	38.1 ± 0.4
Body Weight (g)			264.2 ± 5	268.3 ± 6.3	302.1 ± 6.9	275.5 ± 21.9	299.4 ± 17.0	287.5 ± 19.5
**After treatment (48 h)**								
Rectal Temperature (°C)					37.3 ± 0.1	36.9 ± 0.3	37.6 ± 0.2	37.5 ± 0.2
Body Weight (g)					287.4 ± 8.3	266.8 ± 21.8	288.3 ± 15.4	279.7 ± 23.4
**After treatment (72 h)**								
Rectal Temperature (°C)					37.0 ± 0.2	36.6 ± 0.4	36.2 ± 0.8	36.9 ± 0.5
Body Weight (g)					274.3 ± 10.5	258.8 ± 21.6	281.3 ± 15.8	277.5 ± 26.6

### Evans Blue study

**Figure 2:**
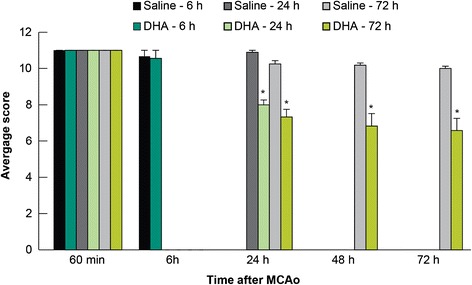
**EB study: Effect of DHA treatment on behavior.** DHA or saline treatments were administered at 1 h after 2 h of MCAo. Total neurologic score (normal = 0, maximal deficits = 12) was measured during MCAo and then at 6, 24, 48 and 72 h after MCAo. DHA-treated animals displayed improved neurologic deficits at 24, 48 and 72 h after MCAo compared to the matching saline group. Values are mean ± SEM; n = 8-12 rats per group. *Significantly different from corresponding saline group (p < 0.05, repeated measures ANOVA followed by Bonferroni tests).

**Figure 3:**
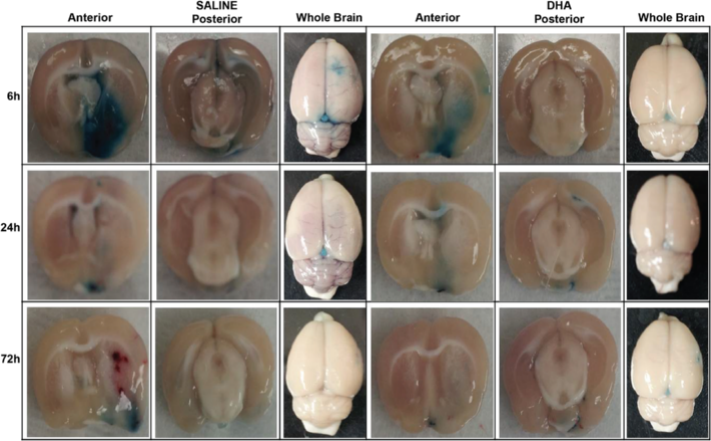
**Representative images of EB extravasation from rats treated with DHA or saline.** Treatments were given at 1 h after 2 h of MCAo and EB was administered at 1 h before rats were sacrificed (at 5 h, 23 h and 71 h). Extensive extravasation of EB is grossly visible in the saline treated rats. Contrarily, substantial inhibition of the dye extravasation was evident in DHA-treated brains.

**Figure 4:**
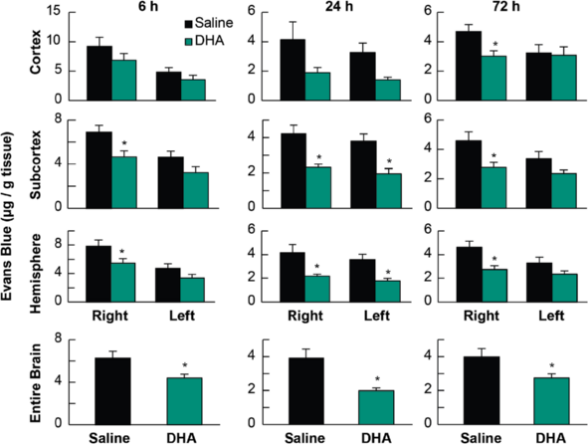
**EB content in rats.** Tissue EB content was measured at 6, 24 and 72 h after MCAo in cortex, subcortex, ipsilateral hemisphere and whole brain in saline- and DHA-treated rats. Treatments were given at 1 h after 2 h of MCAo. EB extravasation was decreased by DHA treatment compared to the corresponding saline group. Values are mean ± SEM; n = 8-12 rats per group. *Significantly different from corresponding saline group (p < 0.05, repeated measures ANOVA followed by Bonferroni tests).

Total neurologic scores were improved in the 24 and 72 h DHA groups compared to the corresponding vehicle-treated groups (Figure 2). There were no differences in the behavioral score between DHA and saline 6 h groups (Figure 2). Tissue leakage of EB was clearly related to cerebral ischemia and presented in Figure 3. Tissue leakage of EB was clearly related to cerebral ischemia, as presented in Figure 3. The brains of the saline-treated rats showed intense blue staining at 6 and 72 h, which were observed predominantly in the ventromedial striatum and the cortex ipsilateral to the MCAo (Figure 3). On the contrary, in the DHA-treated rats, cortical and subcortical regions showed only faint blue staining (Figure 3). DHA treatment significantly reduced EB dye extravasation (Figure 4) into the cortex (at 72 h) and subcortex (at 6, 24 and 72 h). In addition, EB content of the right hemisphere and total EB from the whole brain (Figure 4) were also significantly decreased in all DHA-treated groups compared with the corresponding vehicle group at 6, 24 and 72 h.

### FITC-dextran study

**Figure 5:**
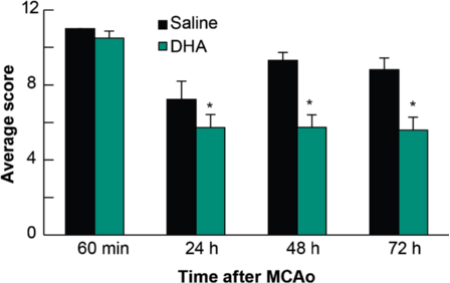
**FITC-dextran study: Effect of DHA treatment on behavior.** DHA or saline treatments were administered at 1 h after 2 h of MCAo. Total neurologic score (normal = 0, maximal deficits = 12) was measured during MCAo and then at 24, 48 and 72 h after MCAo. The degree of behavioral recovery in DHA-treated animals exceeded that of saline group at each time point. Values are mean ± SEM; n = 6-7 rats per group. *Significantly different from corresponding saline group (p < 0.05, repeated measures ANOVA followed by Bonferroni tests).

**Figure 6:**
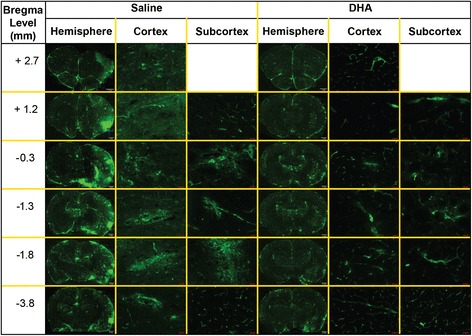
**Representative images of FITC-dextran leakage from rats treated with DHA or saline.** FITC-dextran was intravenously injected at 72 h after MCAo and mosaic images at six bregma levels of entire hemisphere, cortical and subcortical areas were obtained from DHA- or vehicle-treated rats. Diffusive leakage of FITC-dextran was observed within infracted areas in the saline treated rat. In contrast, FITC-fluorescence was dramatically diminished by DHA treatment.

**Figure 7:**
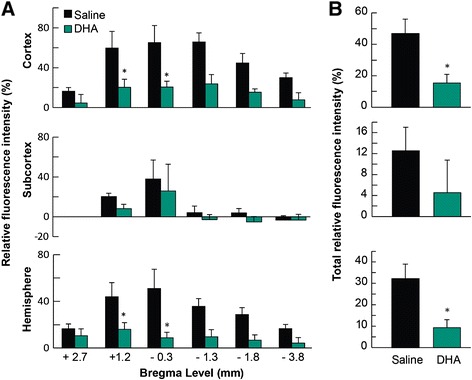
**Effect of DHA treatment on FITC-dextran leakage at 72 h after MCAo. (A)** Relative fluorescent intensity of FITC-dextran at six bregma levels in cortex, subcortex and ischemic hemisphere. **(B)** Total relative fluorescent intensity of FITC-dextran in cortex, subcortex and ischemic hemisphere. FITC-dextran leakage was decreased by DHA treatment compared to the corresponding saline group. Values are mean ± SEM; n = 6-7 rats per group. *Significantly different from corresponding saline group (p < 0.05, repeated measures ANOVA followed by Bonferroni tests).

**Figure 8:**
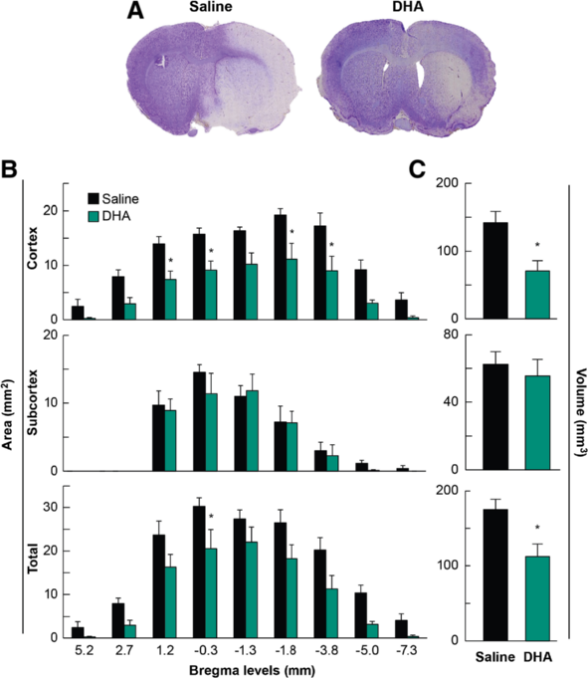
**Histopathology: Effect of DHA treatment on infarct areas and volumes. (A)** Representative computer generated mosaic images of Nissl-stained brain sections from the rats treated with DHA or saline at 72 h after MCAo. DHA showed a smaller cortical and subcortical infarct compared to saline treated animal. **(B)** Infarct areas at standardized bregma levels. DHA decreased cortical and total infarct areas at multiple bregma levels compared to vehicle group. **(C)** Infarct volume in cortex, subcortex and total hemisphere. DHA-treated animals were observed significant reduction of cortical and total infarct volume compared to vehicle-treated animals. Values are mean ± SEM; n = 5-6 rats per group. *Significantly different form corresponding saline group (p < 0.05, repeated measures ANOVA followed by Bonferroni tests: comparison of infarct areas; Student’s *T*-test: comparison of infarct volume).

DHA-treated rats displayed a significantly improved total neurologic score at 24, 48 and 72 h after MCAo compared to the vehicle group (Figure 5). Representative fluorescent images of brains at the six bregma levels from the DHA or vehicle rats at 72 h is presented in Figure 6. Intensive FITC–dextran leakage was observed in the whole ischemic hemisphere, cortex and subcortex in the saline-treated rats. By contrast, FITC-dextran leakage was dramatically reduced by DHA treatment from the microvessels of the ischemic area in both the cortex and subcortex (Figure 6). The alteration of FITC-dextran leakage was measured by the relative FITC-dextran fluorescent intensity to the comparable regions of non-ischemic hemisphere. DHA treatment significantly decreased the relative fluorescence intensity at multiple bregma levels in the cortex and the entire hemisphere compared to the vehicle group (Figure 7A). In addition, total relative fluorescent intensity in the cortex and hemisphere (Figure 7B) was reduced by DHA treatment compared to the vehicle group. Treatment with DHA demonstrated a consistent smaller lesion involving cortical and subcortical regions in the right hemisphere at 72 h after MCAo compared to vehicle group (Figure 8A). DHA treated group significantly reduced the cortical and total infarct areas at multiple bregma levels compared to vehicle treated group (Figure 8B). Moreover, total and cortical infarct volumes in the DHA-treated rats were remarkably decreased compared to saline-treated rats (Figure 8C).

## Discussion

In the present study, we have established a protective effect of DHA treatment against BBB disruption after experimental focal cerebral ischemia. Post-ischemic administration of DHA robustly protected against ischemia-induced BBB disruption, as evidenced by reduced EB extravasation and FITC-dextran leakage, improved the neurological deficits and attenuated infarct volume. Moreover, there were no adverse behavioral side effects observed with DHA administration. Our results suggest that this approach might be adaptable as a therapeutic avenue.

Cerebral ischemia results in rapid accumulation of free fatty acids, including arachidonic acid (ArAc; 20:4) and DHA [10,11]. These fatty acids are released from membrane phospholipids. Both fatty acids are derived from dietary essential fatty acids; however, only DHA, from the omega-3 polyunsaturated fatty acyl chain, is concentrated in phospholipids of various cells of the brain and retina [22]. Synaptic membranes and photoreceptors share the highest content of DHA of all cell membranes. DHA, one of the main structural lipids in the mammalian brain, plays crucial roles in the development and function of brain neurons [10,12]. It is involved in memory formation, promotes neurogenesis both *in vitro* and *in vivo*; and has been implicated in neuroprotection [8,9,23-25]. *In vivo*, the active DHA supply to the brain (provided by the liver through the blood stream) is necessary for cell development and function. It also may play a critical role in conditions where, due to enhanced oxidative stress, the polyunsaturated fatty acyl chains of membrane phospholipids are decreased as a consequence of lipid peroxidation, as occurs in aging, retinal degenerations, and neurodegenerations such as Alzheimer disease [26,27]. In ischemia, there is also loss of brain DHA due to breakdown of enhanced phospholipase A_2_-activated DHA-containing phospholipids [23].

Beneficial effects of DHA in preventing and ameliorating stroke damage have been attributed to generation of the stereospecific derivative, neuroprotectin D1 (NPD1), which is a key survival signaling event leading to neuroprotection [22,28,29]. Recently, we have identified NPD1 following cerebral ischemia-reperfusion in the mouse [28]. NPD1 was found to serve an endogenous neuroprotective role by inhibiting apoptotic DNA damage, upregulating anti-apoptotic and downregulating pro-apoptotic proteins, and also binding toxic peroxides [26,28,29]. We demonstrated that NPD1 synthesis takes place in the ipsilateral side of ischemic brain and peaks at 8 h of reperfusion, and then decreases and is still detectable 25 h after reperfusion [24,28]. Although DHA in the brain can produce NPD1, additional DHA from acute administration is beneficial because lipidomic analysis shows that it potentiates NPD1 synthesis in the penumbra [9,30].

A variety of biologic effects of DHA have been demonstrated with fish or fish oil supplements in humans [31]. They have been found to reduce cholesterol, lower blood pressure, block clot-promoting platelet activation, prevent heart arrhythmias, prevent vascular inflammation and improve vascular function, and protect the heart muscle following a heart attack [32,33]. Epidemiologic studies provide evidence for a beneficial effect of omega-3 fatty acids on manifestations of coronary heart disease and ischemic stroke, particularly with respect to sudden cardiac death in patients with established disease [34]. Clinically important anti-inflammatory effects in humans are further suggested by trials demonstrating benefits of omega-3 fatty acids in rheumatoid arthritis, psoriasis, asthma, inflammatory bowel disorders, osteoporosis, sepsis and cancer [31].

Vascular damage during cerebral ischemia occurs early and it progresses in a biphasic manner [5], contributing to the development of brain edema, hemorrhagic transformation, and worsened clinical outcome in stroke patients [35]. The BBB has a central role in stroke pathogenesis and it is a therapeutic target. Many drugs have been screened to protect BBB permeability integrity and reduce ischemic brain damage by targeting different mechanisms [36]. Unfortunately, so far there are no effective therapeutic interventions for BBB disruption [3]. DHA presence in vascular endothelial cells in the brain suggests that this fatty acid is incorporated into these cells from the systemic circulation [37-39]. DHA in the cell membrane endows fluidity and proper functioning [40]. Furthermore, DHA enriched phospholipids in cellular membranes influences the function of BBB and signaling properties of neurons through fostering a dynamic environment to membrane-associated receptors or enzymes of the BBB compartment [41]. Little is known about the effect of DHA on BBB dysfunction after cerebral ischemia-reperfusion injury. The beneficial effects of pre-treatment with DHA have been reported on BBB disruption, brain edema and inflammatory cell infiltration [42,43]. In contrast, one study showed that post-treatment with DHA (500 nmol/kg, i.p.at 60 min after 90 min of MCAo) exacerbated cerebral ischemic injury and increased BBB permeability in the ischemic cortex at 24 h after reperfusion [44]; note that the DHA dosage applied was 100 times less than in our study. Our study revealed that DHA administration decreased EB extravasation in the ischemic hemisphere at 6 h (by 30%), 24 h (by 48%) and 72 h (by 38%) compared to vehicle-treated rats. In addition, EB extravasation was decreased by DHA treatment in the cortex at 72 h (by 36%) and subcortex at 6 h (by 32%), at 24 h (by 45%) and at 72 h (by 40%) as well as the reduction into the entire brain at 6 h (by 30%), at 24 h (by 49%) and at 72 h (by 31%) compared to the corresponding vehicle groups. We also found that treatment with DHA also suppressed FITC-dextran leakage into the cortex (by 67%) and entire hemisphere (by 71%) compared to the saline-treated animals at 72 h after onset of stroke. In addition, DHA attenuated cortical (by 50%) and total infarct volumes (by 38%) compared to the saline-treated rats at 72 h. These results strongly demonstrate that DHA protects the severely-damaged BBB, accompanied by a relief of abnormal BBB permeability in the delayed ischemic reperfusion damage.

The exact mechanism by which DHA protects the BBB requires further investigation. The beneficial effect of DHA has been shown in a well-controlled animal model of MCAo. In the present study, and in recently published observations [9,13], we have employed a poly-l-lysine coated filament and have found that this technique leads to a 100% incidence of infarction following 2 h of MCAo, predictable size and location of the infarct, and a high degree of inter-animal reproducibility of infarct size (coefficient of variation 8%) [18]. We demonstrated that DHA did not have direct effects on body and cranial temperatures or arterial blood gases because these variables were carefully controlled and did not differ among groups.

## Conclusions

Our results show that DHA is markedly neuroprotective in an experimental stroke model in rats, diminishing BBB damage accompanied with an improvement of behavioral functions and attenuation of the infarct volume. We therefore suggest that this agent offers great promise for developing therapies for cerebral ischemia in patients with acute ischemic stroke.

## References

[1] Ballabh P, Braun A, Nedergaard M (2004). The blood–brain barrier: an overview: structure, regulation, and clinical implications. Neurobiol Dis.

[2] Yang Y, Rosenberg GA (2011). Blood–brain barrier breakdown in acute and chronic cerebrovascular disease. Stroke.

[3] Rosenberg GA (2012). Neurological diseases in relation to the blood–brain barrier. J Cereb Blood Flow Metab.

[4] Warach S, Latour LL (2004). Evidence of reperfusion injury, exacerbated by thrombolytic therapy, in human focal brain ischemia using a novel imaging marker of early blood–brain barrier disruption. Stroke.

[5] Belayev L, Busto R, Zhao W, Ginsberg MD (1996). Quantitative evaluation of blood–brain barrier permeability following middle cerebral artery occlusion in rats. Brain Res.

[6] Huang ZG, Xue D, Preston E, Karbalai H, Buchan AM (1999). Biphasic opening of the blood–brain barrier following transient focal ischemia: Effects of hypothermia. Can J Neurol Sci.

[7] Lo EH, Moskowitz MA, Jacobs TP (2005). Exciting, radical, suicidal: how brain cells die after stroke. Stroke.

[8] Belayev L, Khoutorova L, Atkins KD, Bazan NG (2009). Robust docosahexaenoic acid-mediated neuroprotection in a rat model of transient, focal cerebral ischemia. Stroke.

[9] Belayev L, Khoutorova L, Atkins KD, Eady TN, Hong S, Lu Y, Obenaus A, Bazan NG (2011). Docosahexaenoic acid therapy of experimental ischemic stroke. Transl Stroke Res.

[10] Bazan NG (2007). Omega-3 fatty acids, pro-inflammatory signaling and neuroprotection. Curr Opin Clin Nutr Metab Care.

[11] Bazan NG (2006). The onset of brain injury and neurodegeneration triggers the synthesis of docosanoid neuroprotective signaling. Cell Mol Neurobiol.

[12] Bazan NG, Musto AE, Knott EJ (2011). Endogenous signaling by omega-3 docosahexaenoic acid-derived mediators sustains homeostatic synaptic and circuitry integrity. Mol Neurobiol.

[13] Eady TN, Belayev L, Khoutorova L, Atkins KD, Zhang C, Bazan NG (2012). Docosahexaenoic acid signaling modulates cell survival in experimental ischemic stroke penumbra and initiates long-term repair in young and aged rats. PLoS One.

[14] Sandoval KE, Witt KA (2008). Blood–brain barrier tight junction permeability and ischemic stroke. Neurobiol Dis.

[15] Nagaraja TN, Keenan KA, Fenstermacher JD, Knight RA (2008). Acute leakage patterns of fluorescent plasma flow markers after transient focal cerebral ischemia suggest large openings in blood–brain barrier. Microcirculation.

[16] Uyama O, Okamura N, Yanase M, Narita M, Kawabata K, Sugita M (1988). Quantitative evaluation of vascular permeability in the gerbil brain after transient ischemia using evans blue fluorescence. J Cereb Blood Flow Metab.

[17] Chen B, Friedman B, Cheng Q, Tsai P, Schim E, Kleinfeld D, Lyden PD (2009). Severe blood–brain barrier disruption and surrounding tissue injury. Stroke.

[18] Belayev L, Alonso OF, Busto R, Zhao W, Ginsberg MD (1996). Middle cerebral artery occlusion in the rat by intraluminal suture. neurological and pathological evaluation of an improved model. Stroke.

[19] Belayev L, Busto R, Ikeda M, Rubin LL, Kajiwara A, Morgan L, Ginsbereg MD (1998). Protection against blood–brain barrier disruption in focal cerebral ischemia by the type IV phosphodiesterase inhibitor BBB022: a quantitative study. Brain Res.

[20] Hoffmann A, Bredno J, Wendland M, Derugin N, Ohara P, Wintermark M (2011). High and low molecular weight fluorescein isothiocyanate (FITC)-dextrans to assess blood–brain barrier disruption: technical considerations. Transl Stroke Res.

[21] Jin X, Liu J, Yang Y, Liu KJ, Yang Y, Liu W (2012). Spatiotemporal evolution of blood brain barrier damage and tissue infarction within the first 3 h after ischemia onset. Neurobiol Dis.

[22] Bazan NG (2006). Cell survival matters: docosahexaenoic acid signaling, neuroprotection and photoreceptors. Trends Neurosci.

[23] Bazan NG (2005). Neuroprotectin D1 (NPD1): a DHA-derived mediator that protects brain and retina against cell injury-induced oxidative stress. Brain Pathol.

[24] Belayev L, Marcheselli VL, Khoutorova L, de Turco EB R, Busto R, Ginsberg MD, Bazan NG (2005). Docosahexaenoic acid complexed to albumin elicits high-grade ischemic neuroprotection. Stroke.

[25] Hong SH, Belayev L, Khoutorova L, Obenaus A, Bazan NG (2014). Docosahexaenoic acid confers enduring neuroprotection in experimental stroke. J Neurol Sci.

[26] Bazan NG, Molina MF, Gordon WC (2011). Docosahexaenoic acid signalolipidomics in nutrition: significance in aging, neuroinflammation, macular degeneration, Alzheimer's, and other neurodegenerative diseases. Annu Rev Nutr.

[27] Scott BL, Bazan NG (1989). Membrane docosahexaenoate is supplied to the developing brain and retina by the liver. Proc Natl Acad Sci U S A.

[28] Marcheselli VL, Hong S, Lukiw WJ, Tian XH, Gronert K, Musto A, Hardy M, Gimenez JM, Chiang N, Serhan CN, Bazan NG (2003). Novel docosanoids inhibit brain ischemia-reperfusion-mediated leukocyte infiltration and pro-inflammatory gene expression. J Biol Chem.

[29] Serhan CN, Yacoubian S, Yang R (2008). Anti-inflammatory and proresolving lipid mediators. Annu Rev Pathol.

[30] de Turco EB R, Belayev L, Liu Y, Busto R, Parkins N, Bazan NG, Ginsberg MD (2002). Systemic fatty acid responses to transient focal cerebral ischemia: Influence of neuroprotectant therapy with human albumin. J Neurochem.

[31] Simopoulos AP (2008). The importance of the omega-6/omega-3 fatty acid ratio in cardiovascular disease and other chronic diseases. Exp Biol Med (Maywood).

[32] Das UN (2008). Essential fatty acids and their metabolites could function as endogenous HMG-CoA reductase and ACE enzyme inhibitors, anti-arrhythmic, anti-hypertensive, anti-atherosclerotic, anti-inflammatory, cytoprotective, and cardioprotective molecules. Lipids Health Dis.

[33] Mori TA, Beilin LJ (2004). Omega-3 fatty acids and inflammation. Curr Atheroscler Rep.

[34] Van Bilsen M, Planavila A (2014). Fatty acids and cardiac disease: fuel carrying a message. Acta Physiol (Oxf).

[35] Fagan SC, Hess DC, Hohnadel EJ, Pollock DM, Ergul A (2004). Targets for vascular protection after acute ischemic stroke. Stroke.

[36] Borlongan CV, Rodrigues AA, Oliveira MC (2012). Breaking the barrier in stroke: What should we know? A mini-review. Curr Pharm Des.

[37] Innis SM (2007). Dietary (n-3) fatty acids and brain development. J Nutr.

[38] Rapoport SI, Rao JS, Igarashi M (2007). Brain metabolism of nutritionally essential polyunsaturated fatty acids depends on both the diet and the liver. Prostaglandins Leukot Essent Fatty Acids.

[39] Singh M (2005). Essential fatty acids, DHA and human brain. Indian J Pediatr.

[40] Lukiw WJ, Bazan NG (2008). Docosahexaenoic acid and the aging brain. J Nutr.

[41] Farkas E, de Wilde MC, Kiliaan AJ, Luiten PG (2002). Systemic effects of dietary n-3 PUFA supplementation accompany changes of CNS parameters in cerebral hypoperfusion. Ann N Y Acad Sci.

[42] Cao D, Li M, Xue R, Zheng W, Liu Z, Wang X (2005). Chronic administration of ethyl docosahexaenoate decreases mortality and cerebral edema in ischemic gerbils. Life Sci.

[43] Pan HC, Kao TK, Ou YC, Yang DY, Yen YJ, Wang CC, Chuang YH, Liao S, Raung SL, Wu CW, Chiang AN, Chen CJ (2009). Protective effect of docosahexaenoic acid against brain injury in ischemic rats. J Nutr Biochem.

[44] Yang DY, Pan HC, Yen YJ, Wang CC, Chuang YH, Chen SY, Lin SY, Liao SL, Raung SL, We CW, Chou MC, Chiang AN, Chen CJ (2007). Detrimental effects of post-treatment with fatty acids on brain injury in ischemic rats. Neurotoxicology.

